# Updating the assumptions on the impact of household water, sanitation and hygiene interventions on diarrhea morbidity in young children

**DOI:** 10.7189/jogh.12.08003

**Published:** 2022-03-12

**Authors:** Christa L Fischer Walker, Neff Walker, Robert E Black

**Affiliations:** Department of International Health, Bloomberg School of Public Health, Johns Hopkins University, Baltimore, Maryland, USA

## Abstract

**Background:**

The Lives Saved Tool (*LiST*) is a publicly available and widely used model used to estimate the impact of scaling up interventions on maternal and child health. A strength of the model is that it is continuously updated with country-specific information about intervention coverage, risk factors and causes of death. This paper reports an updated review and meta-analysis on the efficacy of water, sanitation and hygiene (WASH) interventions in reducing diarrhea morbidity among children under the age of five years.

**Methods:**

We updated previous *LiST* systematic reviews for improved WASH interventions according to standard *LiST* criteria. We sought to identify more recent WASH studies to update *LiST* efficacy estimates for each WASH intervention on diarrhea morbidity. In addition, we conducted a search to identify studies that reported an effect size for combined improved WASH interventions. For interventions where we found new studies, we conducted a weighted meta-analysis to produce an updated effect size estimate.

**Results:**

We did not find new studies demonstrating an effect of improved water source alone on diarrhea morbidity among children under 5 years of age. For improved sanitation, we conducted an updated meta-analysis among 4 studies and found no difference between intervention and control arms (weighted mean difference (WMD) = -5% (95% confidence interval (CI) = -11% to 2%). We identified four trials that assessed the effect of combined interventions targeting improved water, sanitation and hygiene. The weighted mean difference also showed no effect on diarrhea morbidity among children under 5 years of age (WMD = -6%, 95% CI = -15% to 4%). Our updated results for handwashing promotion estimate the effects to results in a 17% reduction in childhood diarrhea morbidity (95% CI = 7% to 27%).

**Conclusions:**

Despite widespread acceptance that WASH interventions can improve diarrhea morbidity, the evidence supporting this specifically for children under 5 years of age remains weak. Children interact with the environment in ways that differ from adults and these constant exposures may limit the effect that these WASH interventions can have on diarrhea morbidity.

Diarrhea remains an important cause of death among children under 5 years of age in low- and middle-income countries [1]. Though diarrhea mortality has declined dramatically since the 1980s, further reducing it as a preventable cause of child death will require a targeted approach and a better understanding of the most effective interventions for prevention and treatment [2].

The Lives Saved Tool (*LiST*) is a publicly accessible tool, designed to help policy makers and program planners identify the most effective interventions for reducing cause-specific child mortality [3]. The effect size for each intervention included in *LiST* has been estimated based on peer-reviewed systematic reviews and corresponding meta-analyses [4]. *LiST *strives to include unique effect sizes for each intervention and has historically avoided estimating for a package of interventions, to enable country planners to tailor intervention packages to meet the needs of the country.

The effect sizes for the water, sanitation and hygiene (WASH) interventions used in *LiST* (ie, handwashing with soap, water quality improvement and excreta disposal) were originally reviewed and published in 2010 [5]. Darvesh et al recently updated these in 2017 [6]. Since the literature searches for the Darvesh paper were completed in 2016, three large-scale WASH trials in Kenya, Bangladesh, and Zimbabwe have been published [7-9]. These studies provide much needed information on the effect size of WASH interventions specifically on diarrhea morbidity among children under 5 years of age for both individual and combined intervention effectiveness.

WASH interventions are not often delivered in isolation, rather as a combined WASH package in both research studies and in practice. We sought to update the Cairncross [5] and Darvesh [6] reviews to include studies published after 2016 and generate effect size estimates for individual interventions as well as for a combined WASH intervention approach, which to date has not been included in *LiST.*

## METHODS

We conducted a systematic literature review to identify newly published studies of WASH interventions. We searched PubMed, CINAHL, EMBASE, Cochrane, EconLit, WHO IRIS, and the Global Health Regional indexes for articles published between 1 January 2016 and 30 March 2019. The search strategy was designed using Medical Subject Heading (MeSH) using key words including water, sanitation, hygiene and diarrhea. We de-duplicated articles and screened all titles and abstracts for relevance. All relevant papers were fully reviewed by two independent reviewers to determine suitability per inclusion and exclusion criteria and quality standards for *LiST* review [4]. We also reviewed all included studies from the previous two reviews and rescreened for inclusion and exclusion criteria [5,6]. We reviewed all studies found by Clasen et al [10] and Darvesh et al [6] to identify intervention studies with diarrhea period prevalence, incidence, or more severe diarrhea outcomes among children under 5 years of age and restricted inclusion to studies conducted in low- and middle-income countries.

We included randomized controlled trials (RCTs), cluster randomized controlled trials (cRCTs) and quasi-experimental trials (QE). We included studies that assessed diarrhea morbidity among children under 5 years of age with no greater than 7 days recall by the primary caregiver or diarrhea mortality. We excluded studies conducted in specialized locations or populations such as schools or only among children with a chronic condition. Given the limited number of high-quality studies looking at the effects of water or sanitation interventions alone, we reviewed the evidence base and included studies that considered a combination of WASH interventions as was presented in Clasen et al [10]. If no additional studies were identified, the analysis was not redone and previous reviews were discussed.

**Statistical analysis**

We abstracted the study design descriptors and the diarrhea point prevalence rate based on 7-day recall or incidence based on at least weekly surveillance for all included studies [7-9,11-13]. For studies that did not report a 7-day prevalence, we converted results into a 7-day period prevalence. In a limited number of studies investigators included more than one child under the age of 5 living in the same household in the study. To limit bias, we used a sample size based on the number of households instead of on the total number of children for these studies. For all interventions where we identified new studies for inclusion, we conducted a meta-analysis using a random effects model comparing the mean diarrhea point prevalence rate between intervention and control arms for each comparison group [Review Manager 5.4 (RevMan) Computer Program, The Cochrane Collaboration, 2020] and reported the weighted mean difference (WMD) and 95% confidence interval (CI) for each.

## RESULTS

**Figure 1:**
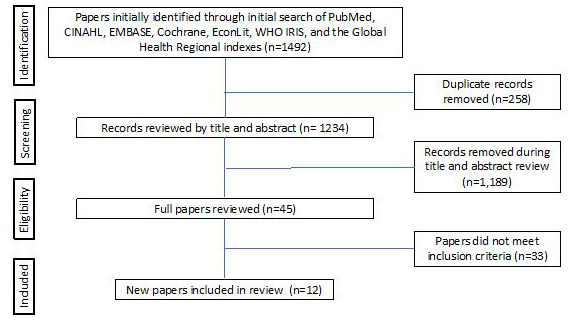
Flow diagram of systematic review paper selection process.

The literature search identified 1234 unique papers published between January 2016 and March 2019 that might contain new data on efficacy of WASH interventions (**Figure 1**). After the title and abstract review, we screened 10 papers for possible inclusion. Three papers met the inclusion and exclusion criteria. We did not identify new studies that met our criteria for interventions on the safe disposal of children’s stools or piped water [6,14,15]. We included interventions on improved water source, improved sanitation in the home/compound, and hand washing each vs a control group.

### Improved water source

In a previous analysis by Cairncross et al (2010), authors identified four studies as improved water “source-based” interventions with outcomes measured among children under five years of age [5]. The relative risk of diarrhea among these studies, as measured by a random effects model, was 0.85 (0.71-1.02). A recently published Cochrane review identified five evaluations of water source interventions, in which improved wells or community water sources were compared to unimproved conditions but where levels of sanitation were not assessed and improvements were not part of the intervention [14]. Four of the studies were controlled before-after evaluations and one was a cluster randomized controlled trial. Overall, there was not a statistically significant reduction in diarrhea and the body of evidence was judged to be of very low quality. It was concluded that there is insufficient evidence to determine if water source interventions alone, such as protected wells, community taps or chlorination/filtration of community water sources, reduce diarrhea incidence [14]. A more recent review found five evaluations of water-source interventions that met their search criteria [6]. The combined meta-analysis indicated no effect of these interventions on the risk of diarrhea (pooled RR: 0.98, 95% CI = 0.73 to 1.32) and the subgroup analysis of interventions involving hand pump wells or chlorination of community water or improved water supply also did not suggest an effect. There were no additional studies found in the updated search with evidence of improved water source alone on diarrhea morbidity and/or mortality.

### Improved sanitation

The Cairncross review identified four small studies of improved sanitation/excreta disposal in China [5]. The interventions were very different and thus not conducive to a pooled effect size. With a lack of quality evidence, the *LiST* estimate put forth referred to a previously published review that combined the effects of pit latrines and toilets [16]. Authors suggested a 36% reduction in diarrhea morbidity, but this effect size included studies that did not specifically focus on children under 5 years of age and a specific description of study design criteria (ie, RCTs or not) was not given.

In later reviews, authors stated that the evidence base for improved sanitation was too weak to estimate an effect size [6,10]. Darvesh et al identified two studies conducted in India that directly addressed the effect of latrine promotion and construction on child health outcomes and neither found an overall effect of child health outcomes specifically focused on children under five years of age [6]. A study evaluated a Total Sanitation Campaign seeking to end open defecation by increasing availability of latrines and changing social norms and behaviors [13]. In this study, toilet use increased by 19% and open defecation decreased by 10%, but there was no effect on childhood diarrhea prevalence (7.4 vs 7.7%, *P* = 0.69) [13]. Clasen and colleagues implemented a rural sanitation campaign with latrine promotion and construction and also observed no difference between intervention and control villages (8.8 vs 9.1%, prevalence ratio = 0.97, 95% confidence interval (CI) = 0.83 to 1.12) [12].

**Figure 2:**
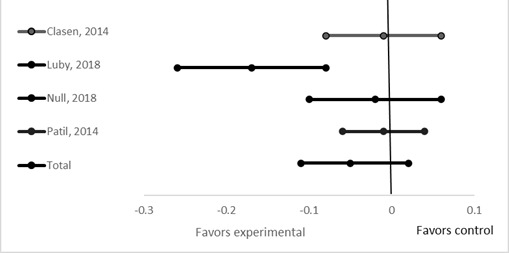
Effect of improved sanitation on 7-day diarrhea prevalence.

We identified two recent studies with data on the effect of sanitation interventions alone on diarrhea morbidity. Luby et al [7] conducted a multi-arm randomized trial in rural Bangladesh to assess various WASH interventions on diarrhea prevalence and child growth among children under 5 years of age [7]. The sanitation arm of the trial provided behavioral change education and latrine construction. In the two years following the intervention, reported diarrhea prevalence was 2.3% less (95% CI = -3.5 to -1.1%) among children living in this intervention households compared with those living in the control households. In a similar study conducted in Kenya, there was no observed difference in childhood diarrhea prevalence between the sanitation and control clusters (diarrhea prevalence in intervention compared to control = -0.3% 95% CI = -3.2 to 2.6) [8]. We conducted a meta-analysis (**Figure 1**) comparing the effects of interventions to improve sanitation alone with a control arm for diarrhea point prevalence among children under 5 years of age for the 4 studies and found no difference between study arms WMD = -5% (95% CI = -11% to 2%) (**Figure 2**).

### Handwashing

**Figure 3:**
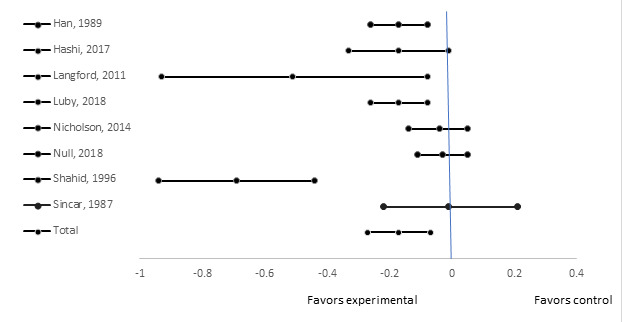
Effect of handwashing promotion on 7-day diarrhea prevalence.

Darvesh et al (2017) recently updated the systematic review on the effect of handwashing with soap on diarrhea indicators and suggested a 27% reduction based on studies focused on children under 5 years of age analysis [6]. We excluded one of the papers included in that review because it included children from 6-15 years in the results, and identified 3 new studies and thus included 8 total studies in the meta-analysis including 5 of the studies from the previous meta-analysis met our inclusion and exclusion criteria and the 3 new studies [7,8,17]. Overall, we estimate a 17% reduction in childhood diarrhea morbidity (**Figure 3**) when effective handwashing promotion interventions are implemented (95% CI = 7%-27%).

### Improved water, sanitation and hygiene packages

**Figure 4:**
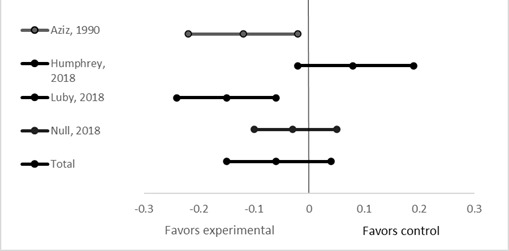
Effect of improved water, sanitation, and hygiene interventions on 7-day diarrhea prevalence.

We identified 4 studies that met our inclusion criteria [7-9,11]. In all studies the intervention group received a package of WASH interventions including improved water source in addition to improved home sanitation facilities and handwashing promotion. In Bangladesh, the children living in villages receiving the rural water, sanitation and hygiene program interventions had fewer reported diarrhea episodes per child year than children in the control villages (2.34 vs 3.12 episodes/child year, *P* < 0.01) [11]. In the large-scale trials in Bangladesh, Kenya, and Zimbabwe (previously described), each included a study arm providing a complete package of both improved water, sanitation and hygiene (handwashing promotion) to randomized villages compared to control villages [7-9]. There was no difference between diarrhea point prevalence in intervention vs control in Kenya and Zimbabwe [8,9]. In Bangladesh the prevalence ratio for the combined WASH intervention was 0.69, 95% CI = 0.53 to 0.90) [7]. The meta-analysis (**Figure 4**) of the four trials showed no effect of water and sanitation on diarrhea rates among children under 5 years of age (WMD = -6%, 95% CI = -15% to 4%) [7-9,11].

## DISCUSSION

We sought to evaluate the evidence and quantify the effect of WASH interventions on diarrhea morbidity among children under 5 years of age. Despite widespread acceptance that improvements in WASH have had an impact on diarrhea morbidity and mortality, the evidence base for these assumptions for children under 5 years remains weak. We applied the same rigorous standards to WASH as for all included LIST interventions and found very few new studies meeting the inclusion/exclusion criteria [4] since the publication by Darvesh et al [6]. *LIST* has included effect sizes for improved water, household access to sanitation, and handwashing since inception, but with the recent publication of 3 large-scale randomized controlled trials an update is warranted. In addition, previous reviews did not include an effect size for combined WASH interventions. We did not find a statistically significant effect of improved sanitation alone or an effect of combined WASH interventions on diarrhea morbidity among young children. However, we did find that handwashing continues to be an intervention reducing diarrhea morbidity among children <5 years of age.

A recent systematic review by Wolfe et al [18], quantified the effects of WASH interventions on diarrheal disease, but included studies that did not specify the effect size on diarrhea among children under 5 years of age and that had cross-sectional study designs [18]. In addition, we found that many previously published WASH studies reported effects of diarrhea for an entire household and did not specifically look at children under 5 years of age. For children under 5 years of age the overall reduction in pathogen exposure may not have been great enough to see a marked difference in diarrhea incidence. More extensive improvements in environmental conditions and exposures, either at the household level [19] or at the community level [9], may be necessary to sufficiently reduce ingestion of diarrhea pathogens to protect highly susceptible young children.

*LiST* includes effect sizes for both improved water source, defined as water from an improved source less than 30 minutes from the home, or piped water in the home. The evidence base we found in our systematic review and included here is for an improved water source and did not include a household piped water connection. We found no evidence that improved water source reduced diarrhea point prevalence. For the initial *LiST* effect size estimates, Cairncross et al reviewed studies that measured an effect of individual interventions, but was limited to available studies with questionable quality (poor randomization, observational studies and excessive recall periods for diarrhea) [5]. Given this limitation and the availability of new evidence, in the most recent version of *LiST* improved water source (except for household piped water) will no longer have an effect on diarrhea morbidity.

This review did not identify studies that met our inclusion and exclusion criteria for piped water. Though it included imperfect study designs and very limited data *LiST* uses the Wolfe et al [18] review and analysis to estimate efficacy of piped water. Wolfe et al found a modest effect of piped water as compared to water from an unimproved source on diarrhea morbidity (risk ratio = 0.77, 95% CI = 0.63 to 0.93). They also found larger effects when piped water was of higher quality (risk ratio = 0.25, 95% CI = 0.09 to 0.67) and there was a continuous water supply (risk ratio = 0.64, 95% CI = 0.42 to 0.98). Based on Wolfe et al, *LiST* assumes that piped water in the home results in a 23% reduction in diarrhea incidence among children below the age of five. *LiST* does not capture effects of piped water quality or continuity of water supply although interruptions in water supply in low- and middle-income countries that do not have the infrastructure to maintain a consistent water supply have also been shown to lead to increased rates of diarrhea [20,21]. *LiST* users could model the effects of suboptimal piped water by decreasing the efficacy of the intervention to account for inconsistencies in flow or quality. The Wolfe et al [18] analysis also found evidence that some home water filtration and storage systems reduced diarrhea morbidity when compared to an unimproved water source (risk ratio = 0.49, 95% CI = 0.38 to 0.64). We did not do a new review this intervention, thus the efficacy values will remain unchanged in *LiST*.

An improvement in household sanitation typically includes the construction of household/compound latrines in areas where household piped water and flush toilets are not possible. We did not observe an effect of improved household level sanitation on childhood diarrhea despite widespread acceptance that household level sanitation decreases open defecation and thus leads to reductions in diarrhea disease. There are limited data with regard to the safe disposal of children’s stools. We did not find any studies that met our inclusion criteria and previous *LiST* analyses were largely based on expert opinion [5,10,22]. Currently *LiST* does not include an effect on diarrhea of providing either improved sanitation facilities or efforts to increase the safe disposal of children stools.

Our analysis did suggest that there is a statistically significant effect of promotion of handwashing with soap on diarrhea incidence. This is in agreement with the previous analysis [6], but our meta-analysis suggests a slightly smaller effect. Our updated analysis included a several large studies that had not been included in Darvesh [7,8,17]. One study that had been included was exclude from our analysis because it included children over 5 years of age [23]. *LiST* should assume that promotion of handwashing with soap can reduce diarrhea incidence by 17%.

Programmatically, several WASH interventions are often implemented in communities simultaneously, thus it is logical to consider a combined effect size. Only 2 of the 4 studies observed a statistically significant benefit. Though we would expect to see a larger effect with the combined WASH interventions, compared to the individual effect of only improved water, access to sanitation or handwashing, this is not what was observed overall in these large-scale studies. These studies are the most rigorously designed studies to date and two contributed data to the sanitation only and improved water only analyses, as well as the combined WASH interventions [7,8].

We conducted a comprehensive literature review with the intention of updating previous reviews. One limitation of this method is the dependency on previous reviews for completeness. We mitigated this limitation by reviewing the numerous published reviews and full Cochrane review reference lists to ensure completeness. Older studies often included participants over the age of 5 and did not present stratified analyses thus leading to their exclusion. Due to the age of the publications, we did not go back to authors to ask for stratified data. This limitation may have led to exclusion of studies that otherwise could have been included. Lastly, the methods and results of the included group of studies were not standard. To enable the inclusion of the greatest number of high-quality studies, we had to convert the results of some studies to 7-day period prevalence rates. For other studies, we handled biases regarding multiple children from the same household by using the household as the sample size in lieu of the number of children.

Despite widespread belief that WASH interventions are critical for the overall reduction in diarrhea morbidity, the evidence does not suggest that classic WASH improvements commonly implemented in LMIC will make much difference in diarrhea morbidity for young children [24]. Children have contact with the environment in ways adults do not, ie, crawling, ingesting objects from the ground, etc [25]. It is clear that protecting children from diarrhea will require more than basic household level improvements to water and sanitation and likely that we will not observe large reductions until more advanced WASH interventions are possible and household level changes can encompass a broader overhaul to prevent fecal exposure and consistent quality and quality of household piped water can be ensured [26]. The Lives Saved Tool relies on the most up-to-date reviews for effectiveness estimates. The estimates provided here will be included in *LiST* to assist policy makers in choosing the most effective interventions in the continued quest to decrease child mortality. Additional rigorously designed studies could provide needed evidence from other populations to continue to expand our understanding of the role of WASH in advancing child health.
